# Exploring the therapeutic potential of “Zhi-Zhen” formula for oxaliplatin resistance in colorectal cancer: an integrated study combining UPLC-QTOF-MS/MS, bioinformatics, network pharmacology, and experimental validation

**DOI:** 10.3389/fmed.2025.1516307

**Published:** 2025-02-26

**Authors:** Yongjing Li, Ke Chen, Qin Li, Qiaoli Liu, Huijie Han, Hui Liu, Songpo Wang

**Affiliations:** ^1^Department of Traditional Chinese Medicine, Shanghai General Hospital, Shanghai Jiao Tong University School of Medicine, Shanghai, China; ^2^Department of Traditional Chinese Medicine, Shuguang Hospital Affiliated to Shanghai University of Traditional Chinese Medicine, Shanghai, China; ^3^Department of Pharmacy, Shanghai General Hospital, Shanghai Jiao Tong University School of Medicine, Shanghai, China

**Keywords:** traditional Chinese medicine, colorectal cancer, chemoresistance, caspase-7, Wnt/*β*-catenin signaling

## Abstract

**Background:**

Chemoresistance is a critical factor compromising the survival of patients with colorectal cancer (CRC). The “Zhi-Zhen” formula (ZZF), a traditional prescription developed by Chinese national medicine masters, has been extensively used in clinical practice to treat gastrointestinal cancer. Notably, ZZF has the potential to enhance tumor sensitivity to chemotherapy. Although previous *in vitro* studies have demonstrated the efficacy of ZZF in overcoming chemoresistance in colorectal cancer (CRC), its precise molecular mechanisms remain poorly understood.

**Materials and methods:**

We used an integrated approach of bioinformatics and network pharmacology to predict the potential active ingredients and targets of ZZF in alleviating chemoresistance. The top five active ingredients identified by degree in the network analysis were validated using mass spectrometry. We then established an oxaliplatin-resistant CRC cell model to explore the potential targets and regulatory mechanisms through which ZZF overcomes chemoresistance at the cellular level.

**Results:**

Network pharmacology and bioinformatics analyses jointly identified 29 active compounds and 13 potential key targets of ZZF, associated with chemoresistance. Among these targets, the differential expression of CASP7 significantly affected the progression-free survival of patients with CRC. We established two oxaliplatin-resistant CRC cell lines and observed an upregulation of CASP7 expression in these resistant cells. Furthermore, ZZF increases the expression and activation of CASP7 in resistant cells, promoting apoptosis, and thereby ameliorating chemoresistance. Additionally, *β*-catenin knockdown led to an upregulation of CASP7 expression, whereas activation of the Wnt/*β*-catenin signaling pathway reduced CASP7 protein levels. ZZF decreases the activity of the Wnt/*β*-catenin signaling pathway by decreasing β-catenin transcription and nuclear localization.

**Conclusion:**

ZZF has potential clinical value in the treatment of chemoresistance in CRC by inhibiting the transcription and nuclear localization of *β*-catenin, thereby increasing the expression of CASP7 and enhancing the apoptotic response in chemoresistant CRC cells.

## 1 Introduction

Colorectal cancer (CRC) is a prevalent malignant tumor with high morbidity (9.6%) and mortality (9.3%) rates among newly diagnosed cancer cases and cancer-related deaths, as reported by Global Cancer Statistics 2022 (1). Population-based data from the National Cancer Institute indicates that the 5-year survival rate of patients with metastatic CRC is approximately 14% (2). Recent advances in targeted therapy and immunotherapy, which have changed the treatment strategies for CRC. The combination of anti-PD-1 and chemotherapy has been the standard first-line treatment for advanced CRC (3). Additionally, chimeric antigen receptor (CAR)-T cell therapy has shown strong anti-tumor effects in CRC. However, treatment efficacy is limited by chemoresistance, the immunosuppressive microenvironment of solid tumors, the lack of tumor-specific antigens, and post-treatment side effects (4). Therefore, chemotherapy remains a key strategy in CRC treatment. It is the primary approach to reduce the risk of recurrence after curative surgery and serves as the cornerstone treatment for metastatic CRC. Oxaliplatin-based chemotherapy regimens (FOLFOX or CAPOX) are the standard first-line treatments for CRC (5). However, chemotherapy often results in acquired chemoresistance, leading to tumor progression and adverse effects on patient survival (6). Overall, acquired chemoresistance is a significant factor contributing to disease recurrence and metastasis, profoundly affecting the prognosis of patients with CRC. Enhancing the sensitivity of CRC to chemotherapy is crucial for improving clinical outcomes.

Chemoresistance arises from a complex interplay of biological processes including multidrug resistance gene expression, apoptosis, epigenetics, and the tumor microenvironment (7). Traditional Chinese medicine (TCM), with its multi-component, multi-target network, and diverse regulatory methods (8), has the potential to enhance the efficacy of chemotherapy. The “Zhi-Zhen” formula (ZZF), developed by the renowned TCM practitioner Zhang Jingren, is composed of herbs including Huang Qi, Nv Zhenzi, Shi Jianchuan, Teng Ligen, Xiang Fu, Ye Putaoteng, and Yi Yiren. Our preliminary studies suggest that ZZF, when combined with chemotherapy, enhances the therapeutic effects on gastrointestinal tumors. As a traditional Chinese herbal formula, ZZF contains numerous herbs and active ingredients with various mechanisms of action. Experimental research has shown that ZZF can reverse chemoresistance in CRC through several mechanisms (9–13): (1) inhibition of P-glycoprotein (P-gp) expression; (2) suppression of IκB-*α* phosphorylation, reducing NF-κB/p65 protein expression; (3) inhibition of the Hedgehog signaling pathway; (4) reduction of P-gp efflux function and inhibition of ERK pathway activity; (5) inhibition of cell proliferation in resistant cells; (6) modulation of M2 macrophage-derived exosomes to alter the immune microenvironment. However, the specific targets and mechanisms of ZZF require further investigation.

This study aims to elucidate the mechanisms and therapeutic targets of ZZF in reversing chemoresistance in CRC through an integrated analysis of bioinformatics, network pharmacology, and cellular experiments. The research aims to provide theoretical insights and empirical data to support the development of future treatment strategies for chemoresistance.

## 2 Materials and methods

### 2.1 Drugs and reagents

Herbs, including Huang Qi, Nv Zhenzi, Shi Jianchuan, Teng Ligen, Xiang Fu, Ye Putaoteng, and Yi Yiren, were procured from Cai Tongde (Shanghai, China). Oxaliplatin, MSAB, Wnt/*β*-catenin agonist 2, and Cell Counting Kit-8 (CCK-8) reagents were sourced from Haoyuan MedChemExpress (Shanghai, China). HyperScript III RT SuperMIX (with gDNA remover) and S6 Universal SYBR qPCR Mix were obtained from NovaBio (Shanghai, China). NE-PER Nuclear and Cytoplasmic Extraction reagents were acquired from Thermo Fisher Scientific (USA). Annexin V-adenomatous polyposis coli (APC)/7-AAD apoptosis kit was purchased from Multi Sciences (Hangzhou, China). Immunostaining Blocking Buffer, DAPI Stain Solution, and Alexa Fluor 594 AffiniPure goat anti-rabbit IgG (H + L) were obtained from Yeasen (Shanghai, China).

### 2.2 Identification of differentially expressed genes

Transcriptome sequencing data and corresponding annotation files from CRC tissues and adjacent normal tissues were acquired from The Cancer Genome Atlas (TCGA). Transcriptome sequencing data of HCT-116 cells with *β*-catenin (CTNNB1) knockdown were obtained from the Gene Expression Omnibus (GEO) database. Raw sequencing data, along with sample and platform information, were downloaded from the GEO database. Data annotation was performed using Python 3.9 (64-bit), resulting in a complete gene expression matrix. Differential expression analysis was conducted using the limma package in R 4.0.3, with genes considered differentially expressed if log2|FC| > 1 and the adjusted *p*-value was <0.05. A volcano plot was generated for data visualization using the ggplot2 package in R 4.0.3.

### 2.3 Screening of candidate components and targets of ZZF

ZZF consists of Huang Qi (Astragali Radix), Nv Zhenzi (Ligustri Lucidi Fructus), Shi Jianchuan (Salvia Chinensis), Teng Ligen (Radix actinidiae), Xiang Fu (Cyperi rhizoma), Ye Putaoteng (Hairy grape stem), and Yi Yiren (Coicis semen). The chemical components of these herbs were obtained from the Traditional Chinese Medicine Systems Pharmacology Database (TCMSP) (14, 15). Additionally, compounds for Ye Putaoteng were obtained from the Chinese Academy of Sciences Chemistry Database and other research (16). These additional compounds were linked to specific TCMSP mol-IDs using their InChIKey and CAS codes. The active ingredients were selected based on the criteria of oral bioavailability (OB) being ≥30% and drug-like properties (DL) being ≥0.18. TCMSP predicts the potential targets of these candidate components. Finally, the protein names of all the targets were converted to gene names using the UniProt protein database.

### 2.4 Identification of chemoresistance targets

Chemoresistance-related targets were identified by searching the GeneCards and OMIM databases using “chemoresistance” and “chemoresistance.” The GeneCards database assigns a score to each target reflecting its relevance to chemoresistance. When many targets were identified, they were filtered these potential targets based on their scores. To compile a comprehensive list of chemoresistance targets, the results from both databases were integrated and the duplicate entries were removed using R (version 4.0.3).

### 2.5 Construction of the network of potential targets of ZZF-herb-active ingredients

To identify candidate therapeutic targets, we analyzed the intersection of targets of the active ingredients of ZZF, DEGs in CRC, and targets related to chemoresistance. Using R (version 4.0.3), we created a Venn diagram to visualize this intersection. Overlapping genes are considered therapeutic targets for overcoming chemoresistance (hereafter referred to as therapeutic targets). Subsequently, we imported the data file into Cytoscape (version 3.7.0) software to construct and visualize the network of potential targets of ZZF-herb-active ingredients of chemoresistance. Furthermore, the network was treated as an undirected network using NetworkAnalyzer in Cytoscape (version 3.7.0).

### 2.6 Gene ontology and Kyoto encyclopedia of genes and genomes enrichment analysis of therapeutic targets

Enrichment analysis of therapeutic targets, including the Gene Ontology (GO) Biological Processes, GO Molecular Function, GO Cellular Components, and Kyoto Encyclopedia of Genes and Genomes (KEGG) Pathway, was performed using the “clusterProfiler” and “enrichplot” packages in R 4.0.3. The results were visualized using the “ggplot2” package to facilitate interpretation in R (version 4.0.3).

### 2.7 Survival analysis

The survival data of patients was downloaded from the TCGA database. The ‘survival’ and ‘survminer’ packages in R (version 4.0.3) were used for survival analysis and visualization.

### 2.8 Preparation of the extracts of ZZF

A total weight of 175 g of Huang Qi, Nv Zhenzi, Yi Yiren, Shi Jianchuan, Teng Ligen, Ye Putaoteng, and Xiang Fu was mixed in a ratio of 6:3:6:6:6:6:2. The mixture was then soaked in 95% ethanol for 20 min and refluxed for 2 h. After filtration, the solution was concentrated with a rotary steamer and converted into a powder by lyophilization, resulting in an ethanol extract of ZZF.

### 2.9 Cell lines and cell culture

Human CRC cell lines, HCT-116 and HCT-8 were acquired from Obio Technology (Shanghai, China) and verified through STR profiling. The cells were cultured in RPMI-1640 medium (Gibco, Carlsbad, CA, United States) supplemented with 10% fetal bovine serum and 1% antibiotics. To establish an oxaliplatin-resistant cell model, HCT-8 and HCT-116 cells were exposed to increasing concentrations of oxaliplatin, ranging from 0.5 μM to 10 μM. The established oxaliplatin-resistant cell lines, HCT-8/LOHP and HCT-116/LOHP were maintained in a complete medium containing 4 μg/mL and 3 μg/mL oxaliplatin, respectively. All cell cultures were maintained at 37°C in a 5% CO_2_ humidified atmosphere.

### 2.10 Real-time quantitative PCR

Total RNA was extracted with the TRIzol reagent and reverse-transcribed into cDNA using HyperScript III RT SuperMIX (with gDNA remover). Real-time quantitative PCR (RT-qPCR) was conducted using the S6 Universal SYBR qPCR Mix according to the instructions of the manufacturer. The primer pairs are shown in Supplementary Table S1. Gene expression levels were calculated as follows:


ΔΔCT=ΔCT_treated–ΔCT_control,Relative expression=2^ΔΔCT.

### 2.11 Drug inhibition analysis

Cells were seeded in 96-well plates at a density of 10,000 cells/well and incubated in fresh medium with or without ZZF for 24 h. Next, the medium was replaced with fresh medium containing various concentrations of oxaliplatin, and the cells were cultured for an additional 24 h. Subsequently, the cells were treated with a medium containing 10% CCK-8 solution for 1 h, and the absorbance at 450 nm was measured using a microplate reader. The inhibition rate was calculated using the following formula:


$$\begin{array}{l} \mathrm{Inhibition}\;\mathrm{rat}=(1-\left(\mathrm{OD\_treated}–\mathrm{OD\_blank}\right)/\\ \left(\mathrm{OD\_control}–\mathrm{OD\_blank}\right)*100\% \end{array}$$

The half-maximal inhibitory concentration (IC50) was determined using GraphPad Prism 8.

### 2.12 Colony formation assay

Cells were diluted to different concentrations, seeded into 6-well plates, and incubated in a growth medium for 48 h. Oxaliplatin was then added to the growth medium and incubated for 10 d. The plates were washed with PBS, fixed with 4% paraformaldehyde, and stained with crystal violet. Finally, images were obtained using a camera and the established colonies were manually counted.

### 2.13 Protein extraction

To obtain total protein, cells were lysed in RIPA buffer containing protease and phosphatase inhibitors. The lysate was then centrifuged, and the supernatant was collected.

Nuclear and cytosolic proteins were extracted using the NE-PER Nuclear and Cytoplasmic Extraction Kit. Briefly, cells were harvested using trypsin and washed with PBS, and the supernatant was discarded to leave the cell pellet. Cells were lysed with Cytoplasmic Extraction Reagent I (CERI), vortexed, and incubated on ice. The supernatant containing cytoplasmic proteins was collected after the addition of Cytoplasmic Extraction Reagent II (CER II) and centrifugation. The remaining pellet was resuspended in Nuclear Extraction Reagent (NER), vortexed, and incubated on ice multiple times to extract nuclear proteins. Following the final centrifugation, the supernatant containing the nuclear proteins was collected. All protein solutions were boiled in 5× loading buffer at 100°C for 10 min.

### 2.14 Western blotting

Proteins were separated by 10% SDS-PAGE and transferred to PVDF membranes. Membranes were blocked with 2% skim milk for 1.5 h at room temperature. Subsequently, the membranes were incubated with primary antibodies overnight at 4°C. The following day, the membranes were incubated with horseradish peroxidase-conjugated secondary antibodies for 1.5 h at room temperature. After washing with TBST, the bands were visualized. The intensities of the bands were analyzed for grayscale values using ImageJ software. The antibodies in this experiment were as follows: Anti-Caspase-7(1:1000, ABclonal), Anti-Bax (1:1000, CST), Anti-Bcl2 (1:1000, CST), Anti-Cleaved-Caspase-7 (1:1000, CST), Anti-Caspase-3 (1:1000, CST), Anti-Cleaved-Caspase-3 (1:1000, CST), Anti-Cleaved-PARP (1:1000, CST), Anti-*β*-catenin (1:1000, CST), Anti-Lamin A/C (1:1000, CST), Anti-GSK-3β (1:1000, CST), Anti-phospho-GSK-3β(Ser9) (1:1000, CST), and Anti-GADPH (1:1000, CST).

### 2.15 Flow cytometry analysis of apoptosis

The cells were seeded in 6-well plates and treated with various reagents for 24 h. Subsequently, the cells were trypsinized, washed twice, and immediately stained with Annexin V-APC and 7-AAD. The apoptosis rate was visualized using the BD Accuri C6 software (BD Biosciences, United States).

### 2.16 Immunofluorescence

A total of 1 × 10^4^ cells were seeded in glass-bottomed cell culture dishes for 24 h before treatment for an additional 24 h. The cells were then fixed with 4% paraformaldehyde for 15 min, washed three times with PBS buffer, and blocked in blocking buffer for 1 h. Subsequently, the cells were incubated with an anti-*β*-catenin antibody (1:100, CST) overnight at 4°C. The next day, cells were washed and incubated with Alexa Fluor 594 AffiniPure goat anti-rabbit IgG (H + L) (1:200, Yeasen) for 1.5 h at room temperature. DAPI staining was performed, and the cells were photographed using a laser scanning confocal microscope (Leica, Italy).

### 2.17 UPLC-QTOF-MS/MS analysis

ZZF (1 mg) was thoroughly dissolved in 1 mL 95% ethanol by ultrasonication for 20 min (50 kHz, 300 W), followed by vortexing and shaking for 30 s. After centrifugation at 4°C and 12,000 rpm for 5 min, the supernatant (800 μL) was used for UPLC-QTOF-MS/MS analysis. Chromatographic separation was performed on a Waters ACQUITY UPLC system using an ACQUITY UPLC BEH C18 column (2.1 mm × 100 mm, 1.7 μm). The mobile phase consisted of eluent A (0.1% formic acid aqueous solution, v/v) and eluent B (acetonitrile). The gradient elution program was as follows: 0–8 min, 10–11% B; 8–14 min, 11–21% B; 14–20 min, 21–27% B; 20–24 min, 27–38% B; 24–31 min, 38–53.5% B; 31–38 min, 53.5–90% B; 38–40 min, 90–10% B. The flow rate was adjusted to 0.4 mL/min, with the column temperature kept steady at 40°C, and an injection volume of 2 μL was used.

The UPLC system was coupled with a Synaptic Q-TOF mass spectrometer. An electrospray ionization source (ESI) was used in both positive and negative ion modes. The MS and MS/MS scan range was m/z 100–1700. The temperatures of the drying gas and sheath gas were 350°C. The capillary voltage was set at 3500 V. The flow rate of the drying gas was 8 L/min. The collision voltage was 150 V and the collision energies were set to 5, 20, 35, and 50 eV. The nebulizer pressure was 50 psi (1 psi, 6.895 kPa).

An Excel table was created to record the compound names, molecular formulae, precise molecular weights of the adducts, and CAS registry numbers (Supplementary Table S2). The table was imported into the PCDL database using PCDL Manager B.08.00. Qualitative Analysis software (version 10.0) was used to identify the components of ZZF based on the chemical structure formula, charge-to-mass ratio, and secondary fragment ion information.

### 2.18 Statistical analysis

Differences between the two groups were assessed using the Student’s t-test. For data involving more than two groups, we used a one-way analysis of variance (ANOVA) followed by Dunnett’s t-test for multiple comparisons. Differences were considered statistically significant at a *p*-value <0.05. Statistical analyses were performed using GraphPad Prism version 8 for Windows. Each experiment was repeated at least three times.

## 3 Results

### 3.1 Caspase-7 presented as a potential target in the mechanism of ZZF against CRC chemoresistance

**Figure 1:**
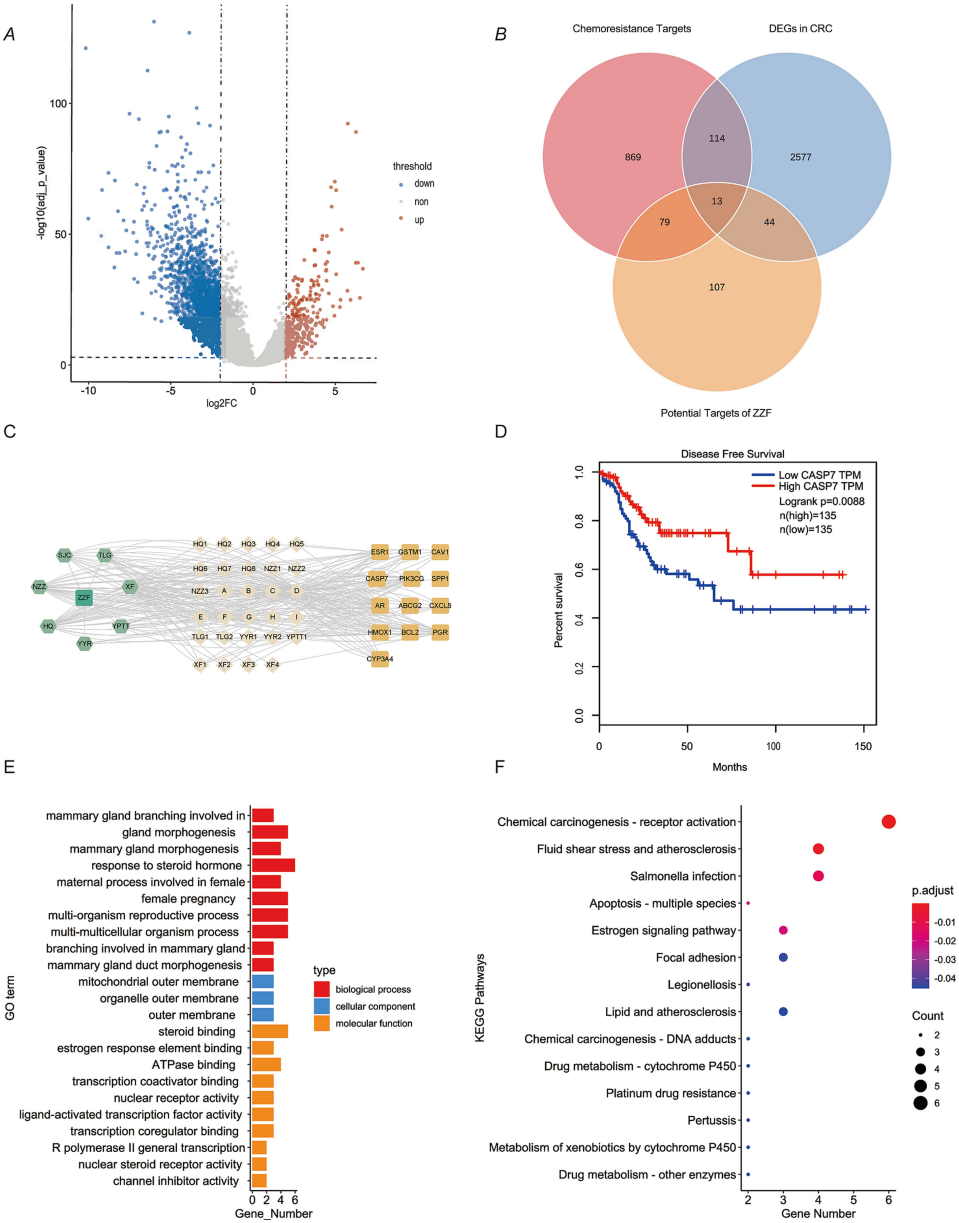
Caspase-7 presented as a potential target in the mechanism of ZZF against colorectal cancer chemoresistance. **(A)** Characterization of differentially expressed genes (DEGs) between colorectal cancer (CRC) and normal tissues in the TCGA database. DEGs were screened with log2 (FC) ≥ 1 and adjusted *p*-value ≤0.01. Blue, significantly downregulated genes; red, markedly upregulated genes. **(B)** Venn diagram shows the overlap of DEGs in CRC, chemoresistance targets, and potential targets of ZZF. **(C)** ZZF-herb-active ingredient-potential targets network diagram. **(D)** Survival analysis of CRC patients grouped based on CASP7 expression level. Data from the TCGA database. **(E)** GO enrichment analysis of therapeutic targets. **(F)** KEGG pathway enrichment analysis of therapeutic targets.

To elucidate how ZZF relieves chemoresistance in CRC, we used bioinformatics and network pharmacology tools in a preliminary study. A total of 2,748 DEGs were identified by analysis of the differences in RNA-seq data between CRC tissues and adjacent normal mucosal tissues in TCGA (Figure 1A). An aggregate of 1,074 chemoresistance-related genes were identified in the databases. By intersecting these genes, we identified 127 potential chemoresistance targets. Additionally, we identified 42 potential active ingredients and 243 potential targets of ZZF from various databases. Intersecting these with 127 chemoresistance targets resulted in 13 potential targets in chemoresistant CRC (Figure 1B). Based on these data, we constructed a ZZF-herbs-active ingredients-potential targets network, comprising 50 nodes (one formula, seven herbs, 29 potential ingredients, and 13 therapeutic targets) and 280 edges (Figure 1C). Network analysis ranked the top five active ingredients by degree, which was validated by mass spectrometry (Table 1; Supplementary Figure S1). To further explore the mechanism of ZZF, we conducted analyses for GO and KEGG enrichment to investigate the common biological functions of the 13 therapeutic targets (Figures 1E,F). We performed KEGG enrichment analysis on the candidate genes and identified several pathways associated with tumorigenesis and drug metabolism. Notably, the “Platinum drug resistance” pathway was enriched among those. A deeper analysis of this pathway revealed the inclusion of BCL2 and GSTM1, both of which are involved in. Furthermore, we found several genes in this pathway closely related to apoptosis, including CASP3, CASP8, CASP9, and BAX. However, the role of CASP7 was not prominently represented. In line with this, Patients with low caspase-7 expression had higher progression-free survival in survival analysis (*p* = 0.0088) (Figure 1D). These findings suggest that caspase-7 is a crucial target for the anti-chemoresistant effect of ZZF in CRC.

**Table 1:** Mass spectrometry identification of the top five ingredients in the ZZF-herb-active ingredient-potential target network.

Sheared Name	Chemical name	Degree	Formula	CAS	RT (min)	Adduct [M + H]+	MS/MS fragments
D	Quercetin	90	C15H10O7	117–39-5	5.328	303.0445	229.0471, 165.0189, 153.0184
C	Kaempferol	42	C15H10O6	520–18-3	6.914	287.0542	213.0546, 153.0167, 135.0360, 115.0542
F	Beta-sitosterol	30	C29H50O	83–46-5	11.974	415.2104	119.0853
E	Luteolin	16	C15H10O6	491–70-3	6.911	285.0385	151.0030, 133.0286
B	Isorhamnetin	12	C16H12O7	491–70-3	-		

### 3.2 ZZF enhanced the apoptotic response of oxaliplatin-resistant cells by upregulating the expression and activation of caspase-7

**Figure 2:**
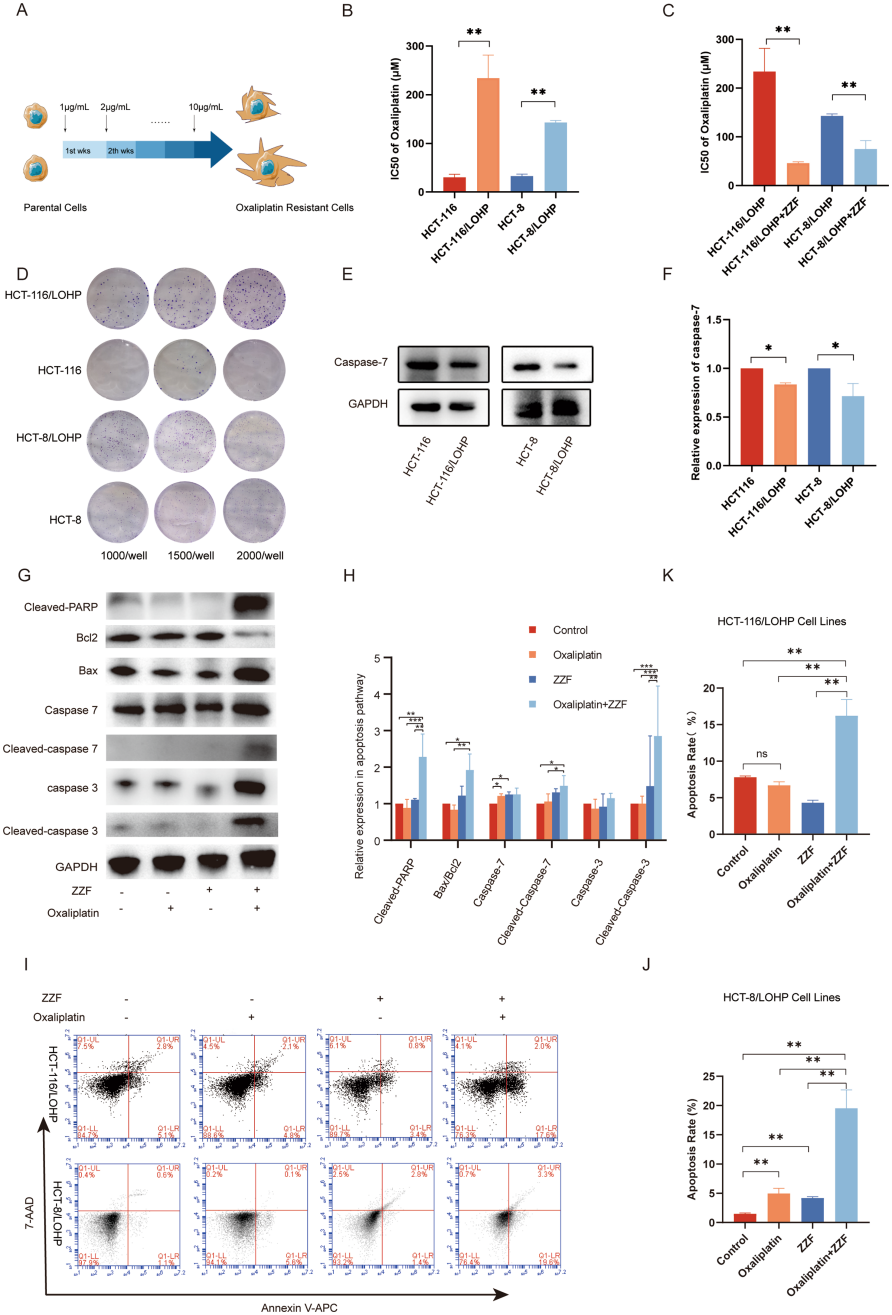
ZZF enhanced the apoptotic response of oxaliplatin-resistant cells by upregulating the expression and activation of caspase-7. **(A)** Schematic diagram of the construction of the oxaliplatin-resistant cell model, HCT-8/LOHP and HCT-116/LOHP, with “wks” denoting weeks. **(B)** The IC50 values of oxaliplatin in parental and resistant cell lines were calculated using GraphPad Prism 8. **(C)** The IC50 of oxaliplatin in drug-resistant cell lines with or without ZZF pretreatment. **(D)** Colony formation assays of the parental and oxaliplatin-treated resistant cell lines. **(E,F)** Expression levels of caspase-7 in parental and resistant cell lines. **(G,H)** Expression levels of apoptosis-related proteins of HCT-116/LOHP in different groups. **(I–K)** Flow cytometry analysis of apoptosis rate of oxaliplatin-resistant cell lines in different groups. *p < 0.05, **p < 0.01, ***p < 0.001 (*n* = 3); ns, no significance (*n* = 3).

With the aim of ascertaining the role of caspase-7 in ZZF’s effects on chemoresistance, we established two oxaliplatin-resistant CRC cell lines, HCT-116/LOHP and HCT-8/LOHP, by sequential drug administration (Figure 2A). The IC50 of oxaliplatin was significantly higher in HCT-116/LOHP cells at 234.17 ± 47.3 μM compared to 30.28 ± 6.46 μM in HCT-116 cells (*p* < 0.05). Similarly, HCT-8/LOHP cells exhibited an IC50 of 142.80 ± 4.30 μM, much higher than 32.62 ± 4.37 μM in HCT-8 cells (*p* < 0.05) (Figure 2B). The colony formation assay confirmed that HCT-116/LOHP and HCT-8/LOHP cells exhibited a greater ability to form colonies in the presence of oxaliplatin than the parental cell lines, indicating increased resistance (Figure 2D). Preconditioning with ZZF reduced the IC50 of oxaliplatin in drug-resistant cells (*p* < 0.05) (Figure 2C), suggesting that ZZF could potentiate chemotherapy sensitivity in CRC. Consistent with previous findings that identified caspase-7 as a putative target of ZZF in reversing chemoresistance, we found that the expression of caspase-7 was significantly lower in oxaliplatin-resistant cells compared to parental cells (*p* < 0.05) (Figures 2E,F). Treatment with ZZF reversed this downregulation, leading to an increase in the expression and activation of caspase-7 in resistant cell lines (Figures 2G,H). Consequently, ZZF treatment combined with oxaliplatin resulted in higher caspase-7 expression and activation, which increased apoptosis in oxaliplatin-resistant cell lines (Figures 2I–K).

### 3.3 ZZF regulated caspase-7 expression by the upstream Wnt/*β*-catenin pathway

**Figure 3:**
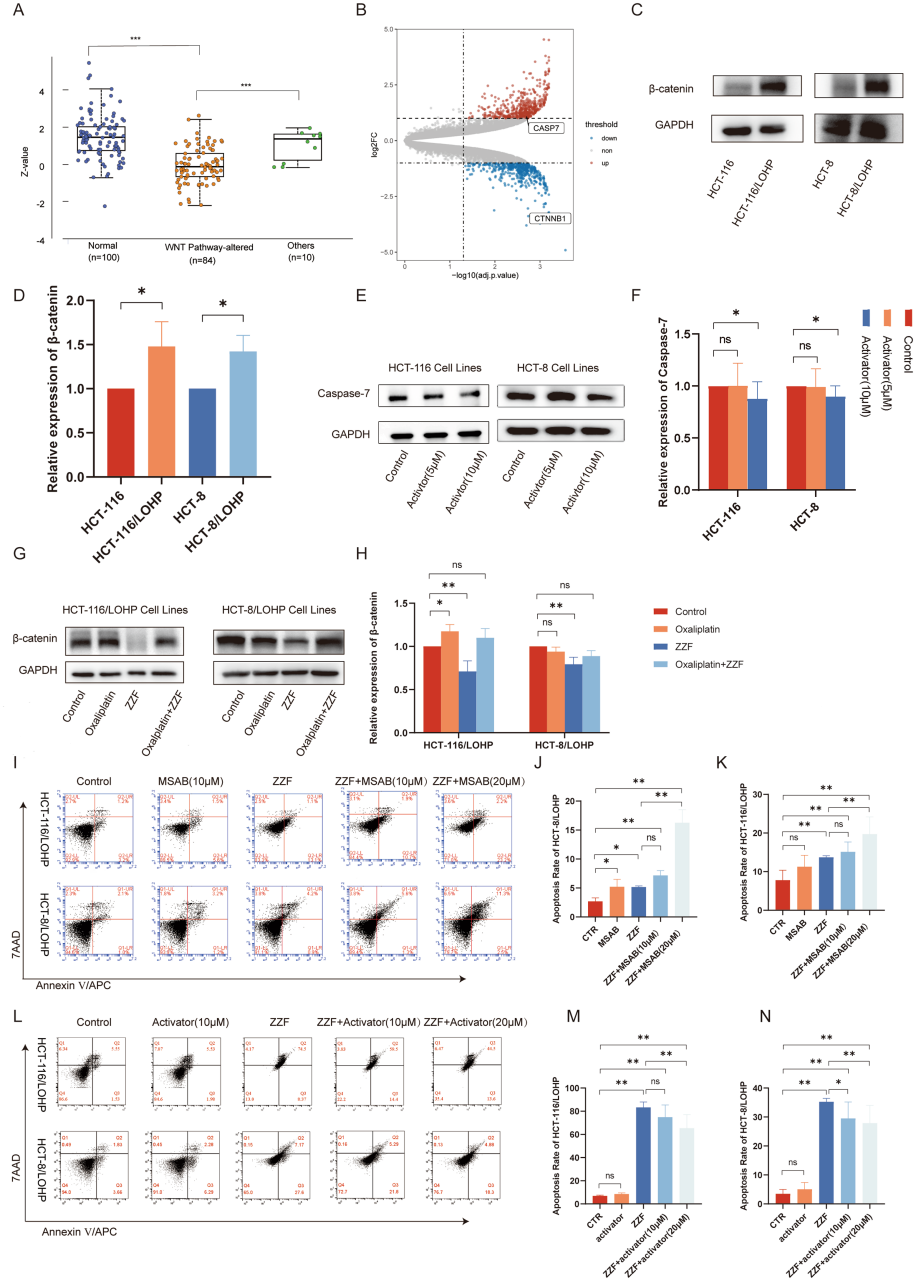
ZZF regulated caspase-7 expression by the upstream Wnt/β-catenin pathway. **(A)** Proteomic data for CASP7 were retrieved from the UALCAN database by selecting the CPTAC colon cancer dataset. Expression profiles were analyzed under the “Total Protein” section based on Wnt pathway status, and a Jitter Plot visualization was generated. Statistical data, including *p*-values, were obtained and used for result interpretation. **(B)** DEGs between wild-type HCT-116 cells and β-catenin knockdown HCT-116 cells in GSE87429. DEGs screened with log2(FC) ≥ 1 and adjusted *p*-value ≤0.01. **(C,D)** β-expression between drug-resistant and parental cell lines was detected by Western blotting (WB). **(E,F)** Effect of Wnt/β-catenin agonist 2 on caspase-7 expression in parental cell lines. **(G,H)** Effect of different treatments on the expression level of β-catenin in drug-resistant cell lines. **(I–K)** Resistant cells were treated with different agents, and flow cytometry was used to investigate the effect of MSAB on ZZF and oxaliplatin-induced apoptosis. The groups were as follows: CTR, the group treated with oxaliplatin alone; MASB, the group treated with both MSAB and oxaliplatin; ZZF, the group treated with ZZF and oxaliplatin; ZZF + MASB represents, the group treated with varying concentrations of MSAB, ZZF, and oxaliplatin in combination. **(L–N)** Flow cytometry analysis of the effect of Wnt/β-catenin agonist 2 on apoptosis induced by the combination of ZZF and oxaliplatin in the resistant cell lines, with the grouping method consistent with figures **(I–K)**. *p < 0.05, **p < 0.01 (*n* = 3); ns, no significance (*n* = 3).

To further explore the upstream pathways through which ZZF regulates CASP7-expression, we searched the UALCAN database using the keyword “CASP7,” and subsequently selected the colon cancer dataset for further analysis. The results revealed that activating Wnt/*β*-catenin signaling led to a significant reduction in caspase-7 expression (*p* < 0.05) (Figure 3A). Conversely, the knockdown of *β*-catenin, a vital component of the Wnt/β-catenin pathway (17), significantly elevated the transcript level of caspase-7 (FC = 2.15, *p* < 0.05) (Figure 3B). Western blotting results indicated higher expression of *β*-catenin in HCT-116/LOHP and HCT-8/LOHP than parental cells (*p* < 0.05) (Figures 3C,D), along with the observed downregulation of caspase-7 in chemoresistant cells. Furthermore, activators of the Wnt/*β*-catenin signaling pathway were observed to downregulate caspase-7 expression in HCT-116 and HCT-8 (*p* < 0.05) (Figures 3E,F). These findings suggest that the Wnt/*β*-catenin pathway is an upstream regulator of CASP7 expression. Moreover, ZZF downregulated *β*-catenin expression in chemoresistant cells (*p* < 0.05) (Figures 3G,H). Flow cytometry results further demonstrated that ZZF and a specific *β*-catenin inhibitor, MSAB, had a synergistic effect, significantly enhancing oxaliplatin-induced apoptosis (*p* < 0.05), respectively (Figures 3I–K). Conversely, Wnt/*β*-catenin agonist 2, a specific β-catenin activator, antagonized the chemosensitization effect of ZZF, inhibiting apoptosis in a concentration gradient (*p* < 0.05) (Figures 3L–N). These results support the role of Wnt/*β*-catenin as an upstream regulator of CASP7-expression in the ZZF mechanism to reverse chemoresistance.

### 3.4 ZZF regulated the Wnt/*β*-catenin pathway by modulating *β*-catenin during transcription and localization

**Figure 4:**
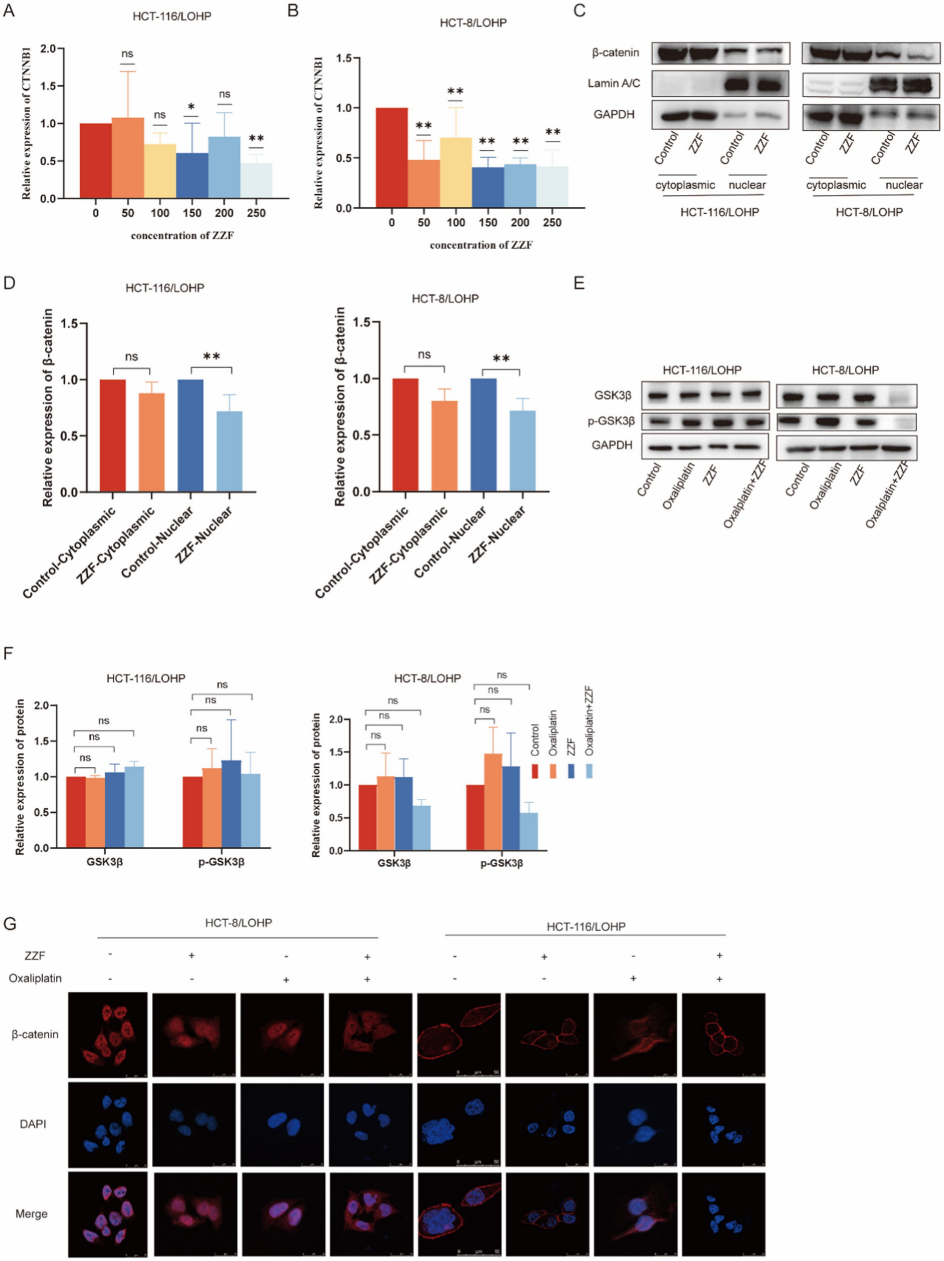
ZZF regulates the Wnt/β-catenin pathway by modulating β-catenin during transcription and localization. **(A,B)** GSK3β and p-GSK3β expression with different treatments in the resistant cell lines. **(C)** ZZF treatment at different concentrations followed by qRT-PCR to assess the mRNA expression level of β-catenin (CTNNB1) in the resistant cell lines. **(D–F)** The resistant cell lines were treated with ZZF for 24 h. Nuclear and cytoplasmic proteins were isolated from the samples, followed by the analysis of β-catenin expression using WB. **(G)** Immunofluorescence image of cells to detect the subcellular localization of β-catenin in the resistant cell lines treated with different treatments. *p < 0.05, **p < 0.01 (*n* = 3); ns, no significance (*n* = 3).

For a more comprehensive understanding of the regulatory mechanisms of ZZF, we investigated the transcription, degradation, and cellular localization of *β*-catenin. The qRT-PCR results indicated that ZZF markedly decreased the mRNA expression of CTNNB1 in chemoresistant cells (*p* < 0.05) (Figures 4A,B). Additionally, GSK3β is a crucial component of the Wnt/β-catenin signaling pathway, contributing to forming the β-catenin degradation complex and promoting β-catenin degradation (18). Western blotting showed that neither ZZF nor oxaliplatin significantly impacted the levels of GSK3β or p-GSK3β (Figures 4E,F). Moreover, the subcellular localization of β-catenin closely links to its function (18). Western blotting revealed that ZZF decreased β-catenin expression, mainly in the nucleus (*p* < 0.05) (Figures 4C,D). Immunofluorescence results demonstrated that the nuclear aggregation of β-catenin was diminished in the ZZF group compared to the control group. Additionally, the nuclear distribution of β-catenin was further decreased in the oxaliplatin combined with the ZZF group versus the oxaliplatin group (Figure 4G). These results indicate that ZZF regulates the Wnt/β-catenin pathway by influencing the transcription and cellular localization of β-catenin.

## 4 Discussion

CRC, with complex pathogenesis and regulatory circuits, stands as one of the most lethal malignancies (19). For decades, oxaliplatin has been the cornerstone chemotherapeutic agent for CRC (20, 21). However, chemoresistance continues to be a significant obstacle that greatly reduces the effectiveness of CRC treatments. Although the mechanisms underlying chemoresistance have been studied to some extent, further investigations are necessary to develop more effective therapeutic strategies (22).

As research on TCM has progressed, scientists have recognized its potential to enhance tumor sensitivity to chemotherapy by modulating cell survival, metabolism, and death through multiple mechanisms (23). ZZF is clinically used to treat CRC owing to its potentiating and detoxifying effects. However, unlike monomolecules, ZZF comprises complex active components that modulate diverse targets, making it challenging to ascertain its pharmacological mechanisms. Network pharmacology and bioinformatics offer tools for investigating the active components and underlying targets of TCM formulae, such as ZZF (24, 25). Utilizing these methodologies, we analyzed and identified the active ingredients and targets of ZZF involved in CRC chemoresistance. We constructed a ZZF–herb–compound–target network and performed mass spectrometry identification of the top five ingredients in the network based on the degree value. The mass spectrometry results demonstrated a certain degree of reliability. However, isorhamnetin, a flavonoid, was not identified in the mass spectrometry ions of ZZF, possibly because of its very low water solubility than quercetin [<3.5 μg/mL (26) and 2.15 mg/L (27) at 25°C]. Further annotation of the targets revealed that the critical targets of ZZF’s anti-chemoresistance effects in CRC were related to platinum-based chemoresistance. This suggests that ZZF regulates platinum resistance through these specific targets. In this study, we established an oxaliplatin-resistant cell model and confirmed the chemosensitizing effects of ZZF in drug-resistant cells.

Additionally, survival analysis identified CASP7, a member of the caspase family involved in apoptosis signaling pathways (28), as a potential target for ZZF to overcome chemoresistance in CRC in our study. The caspase family of proteases is essential for the execution of apoptosis, which is the process of programmed cell death. In both the extrinsic and intrinsic pathways of apoptosis, caspase-3 and caspase-7 are hydrolytically activated and processed by effector cysteine asparaginases to promote the degradation of cellular components and execute cell death (29). Chemoresistance is associated with the evasion of apoptosis through the downregulation of caspase-3 (30). XIAP, belonging to the inhibitor of apoptosis (IAP) protein family, inhibits the activity of caspase-9, caspase-7, and caspase-3, thereby preventing cell death. XIAP is highly expressed in various chemoresistant cancer cells (31, 32). The XIAP/caspase-7 complex has been linked to chemoresistance in caspase-3-deficient breast cancer (33). Inhibition of this complex and reactivation of caspase-7 can sensitize cancer cells to chemotherapy-induced apoptosis (34, 35). Conversely, caspase-7 deficiency confers resistance to endotoxin-induced lymphocyte apoptosis and increases the tolerance of chicken lymphoma cells to chemotherapeutic agents (36). Therefore, reactivating caspase-7 may be a practical approach to overcome chemoresistance. However, the specific mechanism through which caspase-7 affects CRC chemosensitivity remains unclear. Notably, we verified the difference in the expression of caspase-7 between the resistant and parental cell lines. These findings revealed a significant downregulation of caspase-7 in drug-resistant cells, indicating that cancer cells proactively decreased the levels of caspase-7 to gain tolerance to apoptotic stimuli. Moreover, treatment with ZZF in combination with oxaliplatin upregulated caspase-7 expression, ultimately increasing apoptosis in drug-resistant cells. These results suggest that ZZF modulates caspase-7 expression and restores the sensitivity to chemotherapeutic agents.

The Wnt/*β*-catenin signaling pathway is essential for both embryogenesis and maintaining adult tissue homeostasis (37). This pathway regulates the proliferation and differentiation of intestinal epithelial cells (38) and is intricately involved in the initiation and advancement of CRC (39). Aberrant signaling of the Wnt/*β*-catenin pathway has been implicated in chemoresistance in several tumors (40, 41). The β-catenin, a key molecule in the Wnt/*β*-catenin signaling pathway, also functions as an adhesion junction protein (42), but when APC proteins are mutated, *β*-catenin accumulates in the cytoplasm and then shuttles to the nucleus, where it acts as a transcriptional cofactor interacting with TCF family members (e.g., TCF4) to regulate target gene expression (43). Dominant negative TCF/LEF induced the expression of caspase proteins, suggesting that the Wnt/*β*-catenin signaling pathway may participate in the regulation of caspase-7 (44). This hypothesis is corroborated by observations of a negative correlation between *β*-catenin and caspase-7 in CRC, and the knockdown of *β*-catenin upregulates the transcription of caspase-7. Furthermore, activation of the Wnt/β-catenin signaling pathway induced the downregulation of caspase-7 expression. These results indicate that the Wnt/β-catenin signaling pathway is an upstream regulator of caspase-7 expression. From the perspective of cellular function, inhibitors and activators of the Wnt/*β*-catenin signaling pathway exhibited significant interactions alongside ZZF and directly influenced ZZF-induced sensitization of resistant tumor cells to apoptosis. This implies that ZZF can regulate the expression level of caspase-7 by affecting the Wnt/*β*-catenin signaling pathway, thereby enhancing sensitivity for CRC chemotherapy.

The Wnt /*β*-catenin signaling pathway, which is regulated by multiple cellular signals, is primarily influenced by the abundance, activity, and subcellular localization of β-catenin. We discovered that the decrease in *β*-catenin expression mediated by ZZF was attributed to reduced transcription instead of increased protein degradation. This regulatory mechanism may circumvent the activation of other oncogenic factors caused by GSK3β upregulation (45). In addition, ZZF reduced the intranuclear amount and distribution of *β*-catenin. This indicates that ZZF influences the intracellular localization of β-catenin, thereby impeding its functionality.

The Wnt signaling pathway is a complex regulatory network, with the Wnt/β-catenin pathway being the most well-studied component. β-catenin, as a key effector molecule in this pathway, directly influences various biological properties of tumors through its stability and nuclear localization, making it a critical therapeutic target for inhibiting this pathway (46). However, *β*-catenin is considered an “undruggable” molecule due to its inherent challenges in directly targeted by drugs. Current efforts to develop β-catenin inhibitors primarily focus on identifying compounds that can disrupt its interactions with other proteins. For example, the natural compound PKF115-584 has been identified as capable of disrupting the interaction between β-catenin and TCF complexes, thereby inhibiting the proliferation of CRC cells (47). Additionally, Sec62 binds to β-catenin, inhibiting its degradation and enhancing Wnt signaling, which promotes CRC stemness and chemoresistance (48). Despite promising preclinical and clinical results, no drugs targeting the Wnt/β-catenin pathway have been approved (49). Challenges include unknown mechanisms, regulatory factors, and potential safety risks (50). Multi-target strategies offer new directions. ZZF, a traditional Chinese medicine formula, has shown unique multi-target advantages. This study demonstrates that ZZF suppresses *β*-catenin activity, increases Caspase-7 expression, and enhances the apoptotic response in chemotherapy-resistant colorectal cancer cells, overcoming the limitations of single-target therapies and improving chemoresistance.

Although this study delivers valuable insights into the effects of ZZF on ameliorating oxaliplatin resistance in CRC by regulating the Wnt/β-catenin/Caspase-7 signaling pathway, it is essential to acknowledge certain limitations. This study primarily relied on *in vitro* experiments, and the effects of ZZF have not been validated in animal models. The *in vivo* environment is more complex, with factors such as the tumor microenvironment and drug metabolism potentially influencing the efficacy of ZZF. Therefore, the lack of in vivo validation may limit the generalizability of the results. ZZF is a multi-component herbal formula, and variations in ingredient composition across different batches may affect its therapeutic efficacy. Factors such as cultivation, harvesting, and storage conditions can influence the concentration of active compounds, leading to variability in therapeutic outcomes. Future research should focus on optimizing and standardizing its composition to ensure consistent and reliable therapeutic effects. Although a correlation between *β*-catenin and caspase-7 has been observed, its precise mechanism remains unclear. Future research should aim to elucidate this mechanism to understand better how the Wnt/β-catenin pathway regulates caspase-7 and its role in CRC chemoresistance. Additionally, future studies could explore the therapeutic potential of various combinations of chemical components from the ethanol extract of ZZF to determine whether specific combinations or individual components are primarily responsible for its effects and provide new insights for formulation optimization and novel drug development.

## 5 Conclusion

**Figure 5:**
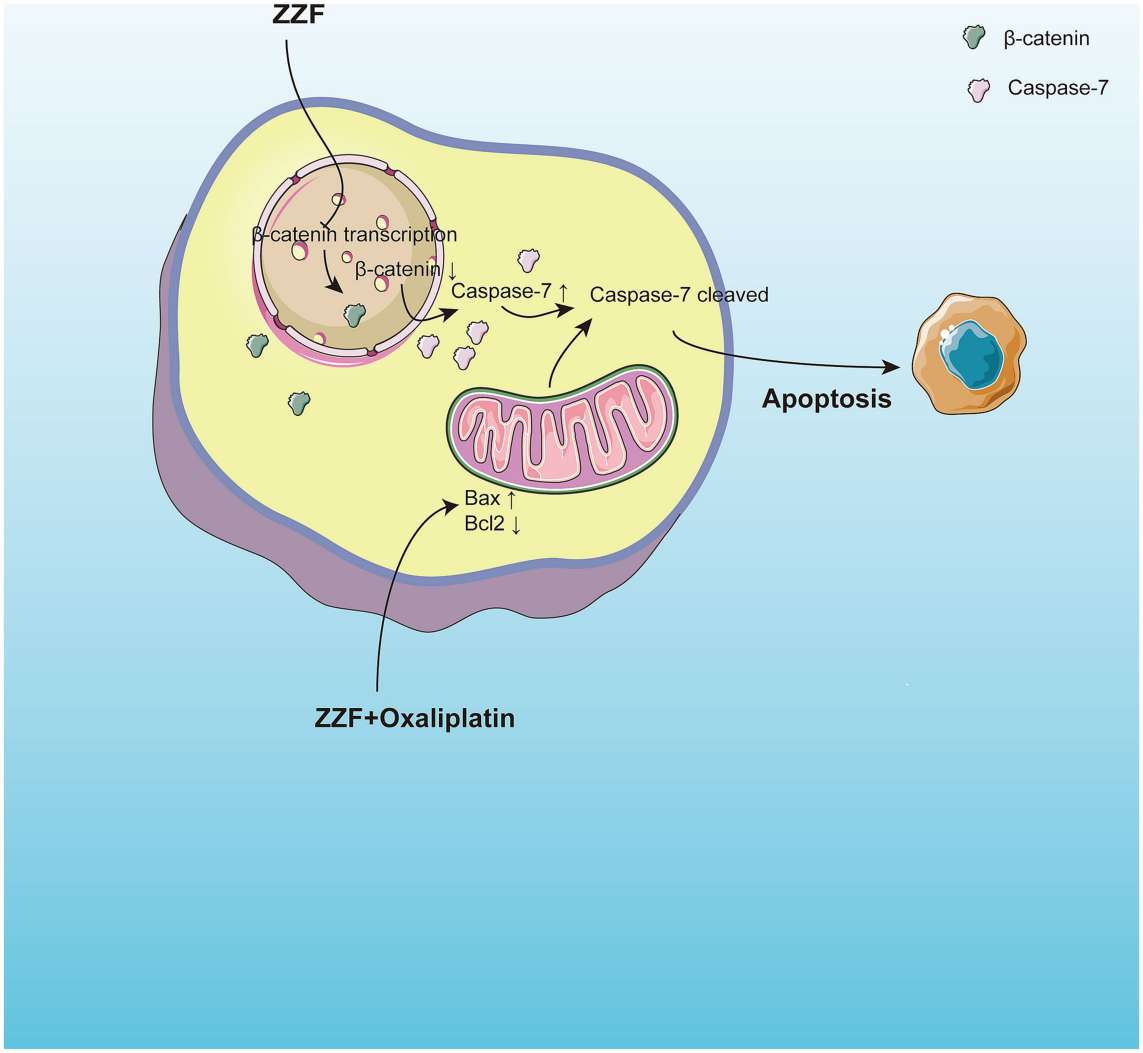
ZZF reinstates apoptotic capacities via regulating β-catenin and caspase-7 in chemoresistant colorectal cancer. ZZF regulates the nonconstitutive expression of caspase-7 and the expression and nuclear localization of β-catenin to influence the sensitivity of chemoresistant CRC cells to apoptosis.

In this study, we utilized bioinformatics and network pharmacology to identify the active components and potential target genes of the traditional herbal formula ZZF involved in overcoming chemoresistance in colorectal cancer. These findings offer a novel perspective on addressing chemoresistance. Validation experiments using an oxaliplatin-resistant CRC cell model demonstrated that ZZF enhances the response of chemoresistant CRC cells to apoptosis by inhibiting the transcription and nuclear localization of β-catenin and increasing the expression of caspase-7 to reverse chemoresistance. Additionally, an analysis of ZZF components highlighted the unique role of traditional Chinese medicine in multi-target, multi-level regulation, emphasizing the need for further research to fully elucidate the molecular mechanisms by which ZZF reverses chemoresistance. Such insights could pave the way for the development of new adjuvant therapeutic strategies for chemotherapy (Figure 5).

## Data Availability

The raw data supporting the conclusions of this article will be made available by the authors, without undue reservation.

## Supplementary material

SUPPLEMENTARY FIGURE S1 Mass spectrometry identification of the top five ingredients in the ZZF-herb-active ingredient-potential target network.
