# Supplementary figures and images for: Exploring the therapeutic potential of “Zhi-Zhen” formula for oxaliplatin resistance in colorectal cancer: an integrated study combining UPLC-QTOF-MS/MS, bioinformatics, network pharmacology, and experimental validation
Source: Front Med (Lausanne). 2025 Feb 26;12:1516307. doi: 10.3389/fmed.2025.1516307 (PMC11897289; doi:10.3389/fmed.2025.1516307)

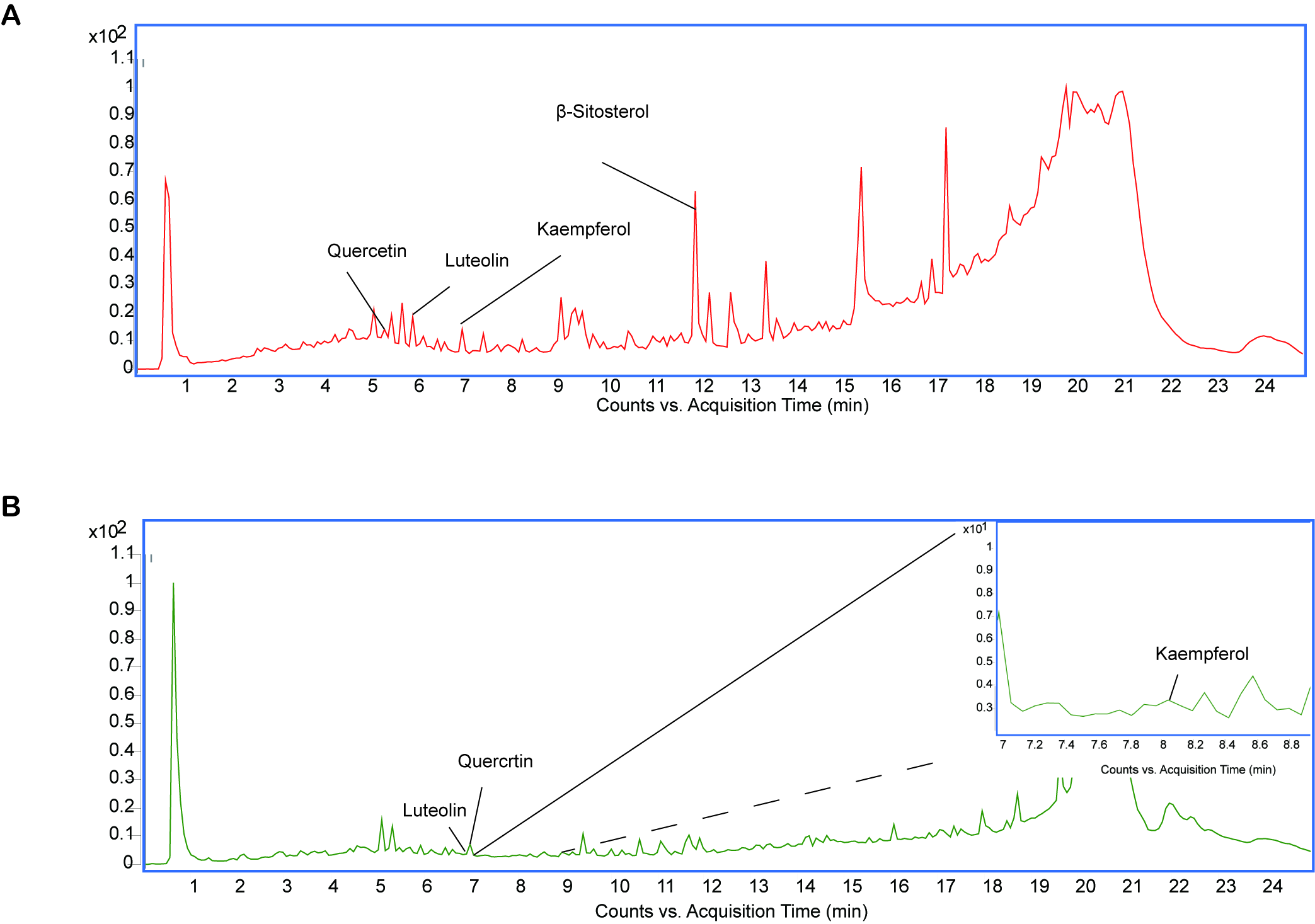

Supplement: SUPPLEMENTARY FIGURE S1 — Mass spectrometry identification of the top five ingredients in the ZZF-herb-active ingredient-potential target network. [file Image_1.TIF]
