# Supplementary material for: Exploring the therapeutic potential of “Zhi-Zhen” formula for oxaliplatin resistance in colorectal cancer: an integrated study combining UPLC-QTOF-MS/MS, bioinformatics, network pharmacology, and experimental validation
Source: Front Med (Lausanne). 2025 Feb 26;12:1516307. doi: 10.3389/fmed.2025.1516307 (PMC11897289; doi:10.3389/fmed.2025.1516307)
Supplement: Supplementary file 4 [file Table_3.DOCX]

Wang Songpo has published several significant articles in core Chinese journals, particularly focusing on the reversal of tumor chemotherapy resistance and the treatment of gastrointestinal diseases through traditional Chinese medicine (TCM). His research often centers on exploring how compound prescriptions, such as Zhizhen Formula, can modulate specific signaling pathways (e.g., Hedgehog, NF-κB, Akt) to reverse tumor drug resistance, revealing the potential application of TCM in enhancing the efficacy of chemotherapy drugs. In addition, he has published experimental studies on the regulation of gastrointestinal diseases, further advancing the theoretical and clinical practice of integrative medicine.

These articles are mainly published in core Chinese journals, such as the "Journal of Traditional Chinese Medicine," "Chinese Journal of Integrated Traditional and Western Medicine on Digestion," and "World Journal of Traditional Chinese Medicine," reflecting his significant contributions and status in the field of TCM research. His work not only provides a critical foundation for the application of TCM in cancer treatment but also opens new avenues for treating gastrointestinal mucosal protection, anti-inflammation, and related diseases.
