# Supplementary material for: Exploring the therapeutic potential of “Zhi-Zhen” formula for oxaliplatin resistance in colorectal cancer: an integrated study combining UPLC-QTOF-MS/MS, bioinformatics, network pharmacology, and experimental validation
Source: Front Med (Lausanne). 2025 Feb 26;12:1516307. doi: 10.3389/fmed.2025.1516307 (PMC11897289; doi:10.3389/fmed.2025.1516307)

Source data Fig. 2E

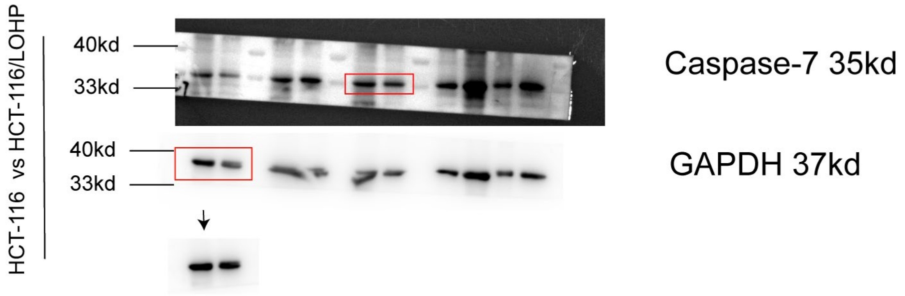

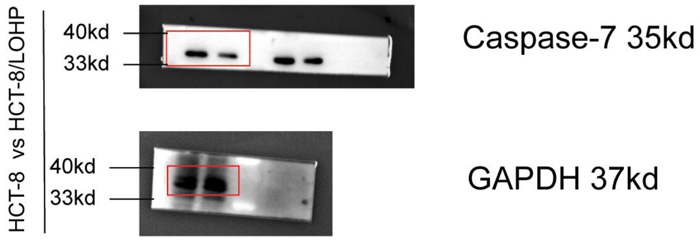

Source data Fig. 2G

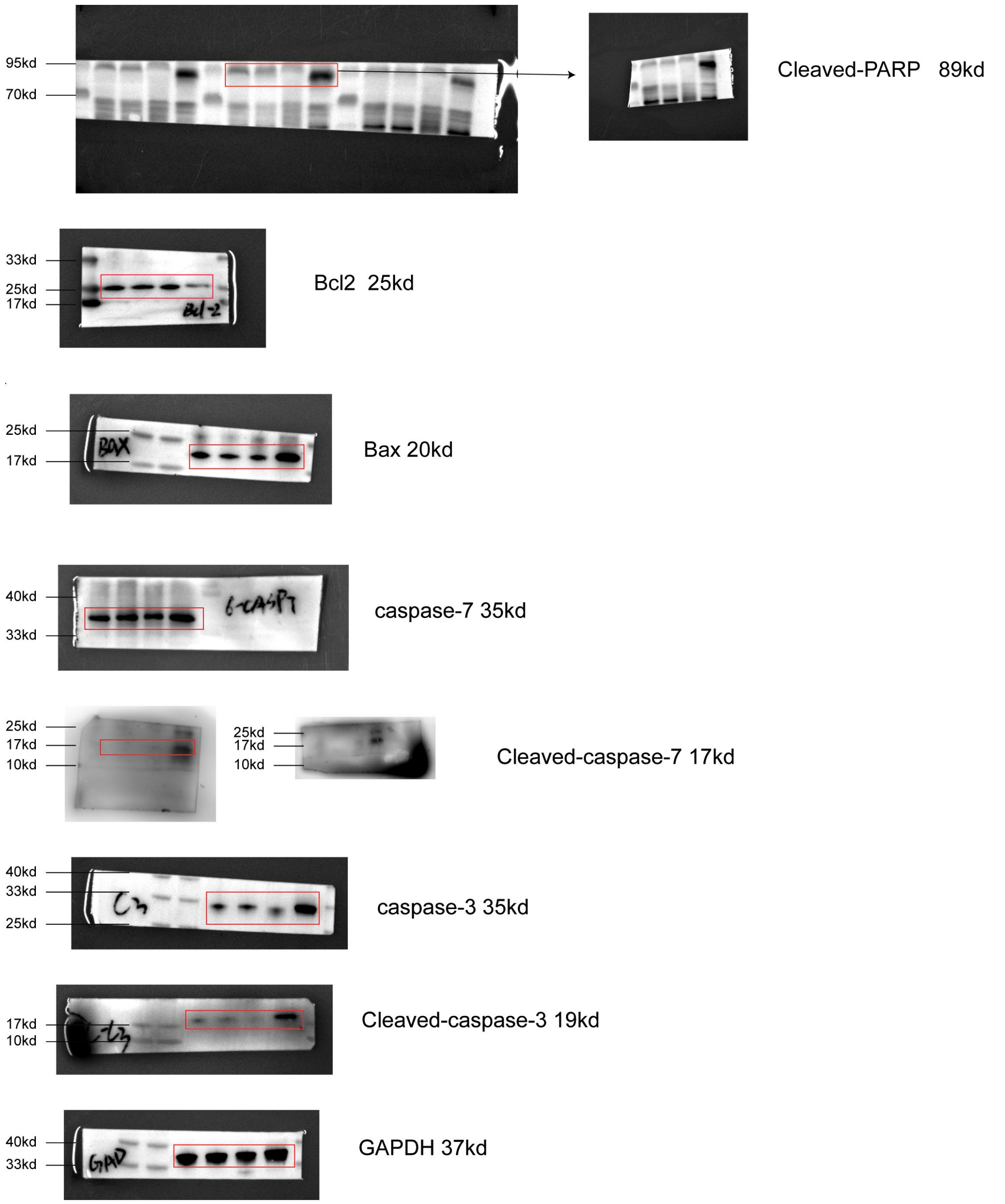

Source data Fig. 3C

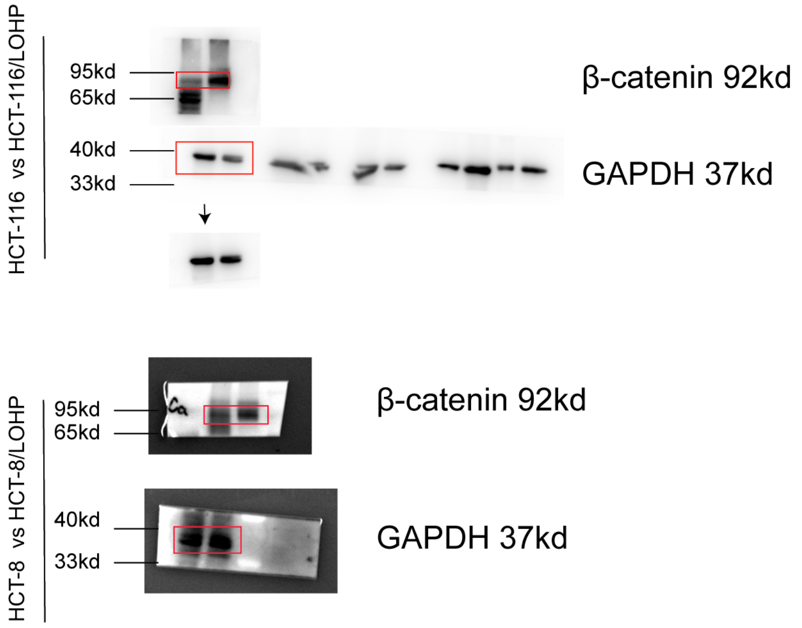

Source data Fig. 3E

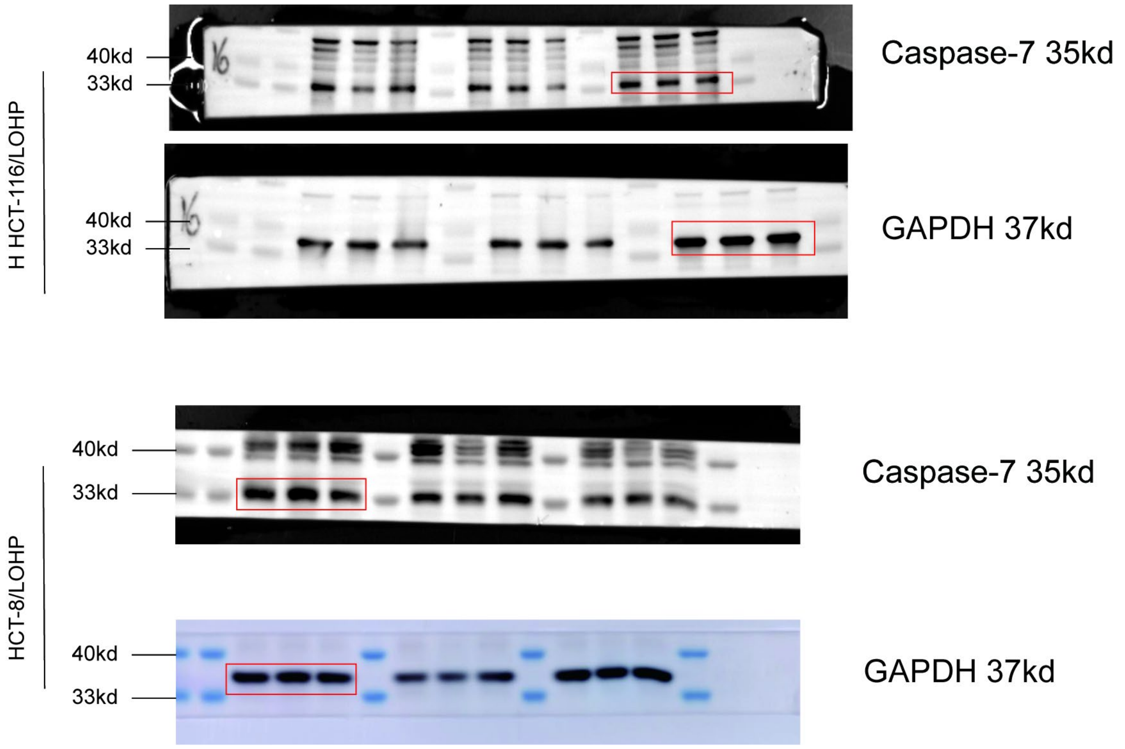

Source data Fig. 4C

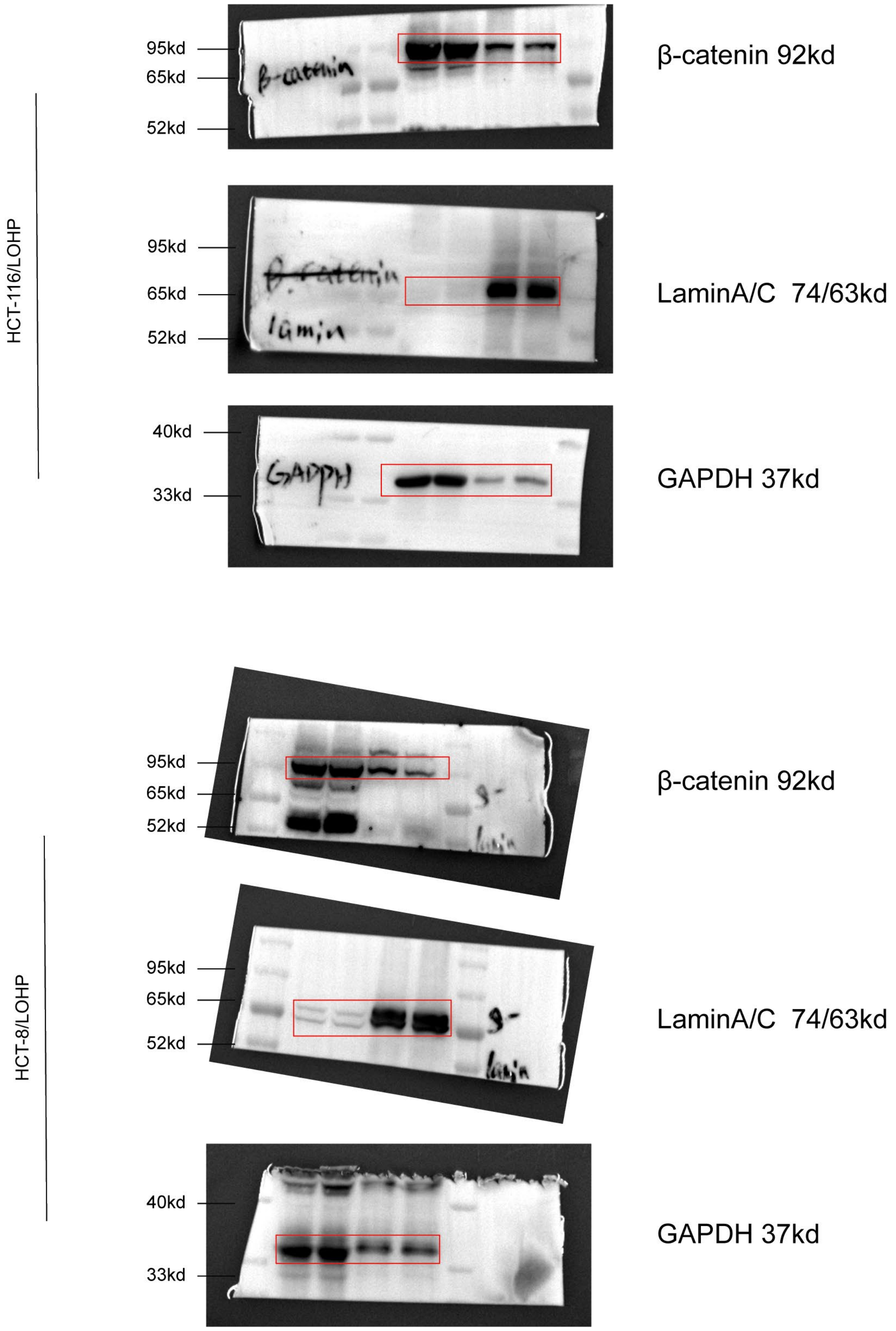

Source data Fig. 4E

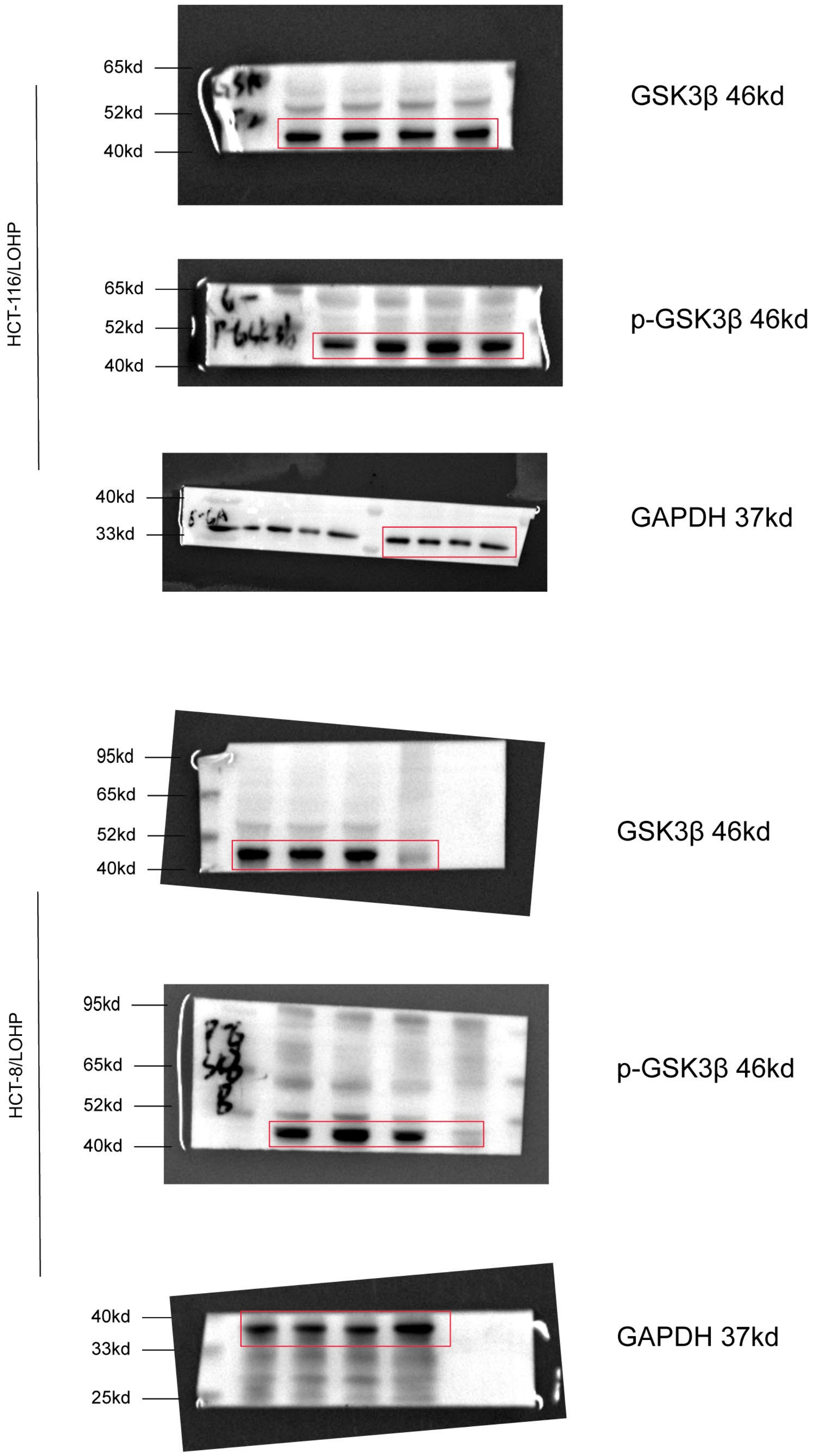

Supplement: Supplementary file 5 [file Data_Sheet_1.PDF]
